# How do postgraduate GP trainees regulate their learning and what helps and hinders them? A qualitative study

**DOI:** 10.1186/1472-6920-12-67

**Published:** 2012-08-06

**Authors:** Margaretha H Sagasser, Anneke WM Kramer, Cees PM van der Vleuten

**Affiliations:** 1 Department of Primary and Community Care, Radboud University Nijmegen Medical Centre, Radboud, The Netherlands; 2 Department of Educational Development and Research, Faculty of Health, Medicine, and Life Sciences, Maastricht University, Maastricht, The Netherlands; 3 King Saud University, Riyadh, Saudi Arabia; 4 University of Copenhagen, Copenhagen, Denmark

**Keywords:** Self-regulation, Workplace-based learning, Postgraduate training, Professional development, Qualitative research methods

## Abstract

**Background:**

Self-regulation is essential for professional development. It involves monitoring of performance, identifying domains for improvement, undertaking learning activities, applying newly learned knowledge and skills and self-assessing performance. Since self-assessment alone is ineffective in identifying weaknesses, learners should seek external feedback too. Externally regulated educational interventions, like reflection, learning portfolios, assessments and progress meetings, are increasingly used to scaffold self-regulation.

The aim of this study is to explore how postgraduate trainees regulate their learning in the workplace, how external regulation promotes self-regulation and which elements facilitate or impede self-regulation and learning.

**Methods:**

In a qualitative study with a phenomenologic approach we interviewed first- and third-year GP trainees from two universities in the Netherlands. Twenty-one verbatim transcripts were coded. Through iterative discussion the researchers agreed on the interpretation of the data and saturation was reached.

**Results:**

Trainees used a short and a long self-regulation loop. The short loop took one week at most and was focused on problems that were easy to resolve and needed minor learning activities. The long loop was focused on complex or recurring problems needing multiple and planned longitudinal learning activities. External assessments and formal training affected the long but not the short loop. The supervisor had a facilitating role in both loops. Self-confidence was used to gauge competence.Elements influencing self-regulation were classified into three dimensions: personal (strong motivation to become a good doctor), interpersonal (stimulation from others) and contextual (organizational and educational features).

**Conclusions:**

Trainees did purposefully self-regulate their learning. Learning in the short loop may not be visible to others. Trainees should be encouraged to actively seek and use external feedback in both loops. An important question for further research is which educational interventions might be used to scaffold learning in the short loop. Investing in supervisor quality remains important, since they are close to trainee learning in both loops.

## Background

Because of the complexity of medical practice and the rapid development of new medical knowledge and skills, it is unrealistic to expect that training programmes can prepare trainees for all the situations they may encounter during their future professional careers. It is therefore important for trainees to learn how to regulate their continuing professional development. According to theory, self-regulation is a deliberate process of professional development including 1) monitoring of and/or retrospective reflection on daily practice, 2) identifying areas of knowledge or skills that have dropped below professional (or personal) standards of practice, 3) seeking appropriate learning opportunities, 4) putting new knowledge/skills into action and 5) re-evaluating one's performance [1,2]. Self-regulation is a complex interactive process involving cognitive, meta-cognitive and motivational aspects [3]. Reflection is important as a powerful tool for making deliberate choices about one’s development [4-6]. Self-regulation may be influenced by learners’ study and learning orientations, as these influence learners’ study goals and the way they plan and organize their studying [7]. Self-regulation is also described in learning styles [8,9]. Self-assessment is important for self-regulation, but several studies have shown that self-assessment alone is ineffective in identifying weaknesses [1,10-12], because of its low correlation with external assessments and its variation with content, context and in what it brings about [13-16]. The purpose of self-assessment should be formative, in other words, it should inform actions to improve practice and self-monitoring aimed at integrating internal and external information [17,18]. Whilst external information is derived from observation, external assessments, et cetera [19,20], internal information stems from cognitive or affective processes [17,21]. Self-assessment should preferably not be a solitary activity, but encompass self-directed assessment seeking. In other words, learners should be challenged to seek reliable and valid external feedback in addition to their self-assessment [12,22].

Postgraduate medical training consists largely of working in clinical practice, which is known to be a powerful learning environment [23]. Today's postgraduate training programmes increasingly incorporate educational elements specifically aimed at stimulating learners’ self-regulation, such as reflection on experiences, formulating learning goals and documentation of learning in a portfolio [24-26]. Self-regulation can also be initiated when learners use information about their progress from external mandatory assessments and progress meetings as input for further learning activities [27]. The way self-regulation may be facilitated and stimulated has been studied in many medical education settings [28-33].

As presented in the literature self-regulation is mainly a theoretical model. We wanted to explore how self-regulation occurs in practice. In the present study, we investigated how postgraduate trainees regulate their learning and how self-regulation is guided by external support. We were specifically interested in how trainees monitor and assess their performance and how they use information from external sources to guide their regulation activities. Additionally, we wanted to gain insight into barriers to and facilitators of self-regulation.

As our aim was to gain in-depth insight into trainees’ learning experiences, we conducted a qualitative study with a phenomenologic approach [34,35] aimed at 1) understanding the role of self-regulation in the learning of postgraduate trainees and 2) gaining insight into barriers to and facilitators of self-regulation and learning. From an expertise development perspective, we also explored whether there was a difference in self-regulation between novice and more advanced trainees [36,37]. Using a qualitative approach, we conducted and analyzed interviews with trainees at different stages of a postgraduate training programme.

## Methods

### Context

The study was performed among trainees of the three-year competency-based postgraduate training programme in general practice (GP) in the Netherlands [38]. In years 1 and 3 of the programme, trainees work in a general practice setting where they are coached and instructed by one supervisor. Year 2 consists of rotations in hospitals, nursing homes and psychiatric outpatient clinics with different supervisors. During years 1 and 3, trainees work four days a week in a general practice and on the fifth day they attend a day-release programme in groups of twelve to fifteen trainees facilitated by two mentors. GP training thus consists mostly of workplace-based learning aimed at connecting trainees’ clinical experiences with theoretical background. Reflection and feedback on experience, assessment and personal development are also important aspects of GP training. As a way to guide their learning, trainees are encouraged to write a learning plan and reflections on their learning for inclusion in their learning portfolio. In the course of the training programme trainees take part in several mandatory external assessments. Trainees’ communication skills are assessed from video recordings of consultations conducted by the trainees (communication video assessment). Knowledge in various medical domains is assessed by multiple choice tests consisting of questions about paper patients (progress knowledge test). Three progress meetings are scheduled every year, in which trainees discuss their development with their mentors and supervisor along the lines of a competency-based assessment framework.

### Design

As our aim was to discover, describe and interpret trainees’ lived experiences, a qualitative study with a phenomenological approach was considered appropriate [34,35]. The phenomenological approach we took is known as new or American phenomenology [34]. New phenomenology questions do not usually seek the prereflexive experience but include thoughts and interpretations of the experience in the data collection and analysis. In new phenomenology analysis focuses on describing participants’ lived experience within the context of culture as opposed for a universal meaning of it. The phenomenon under study is the process of self-regulation GP-trainees apply in practice. We conducted semi-structured interviews with individual trainees [39]. Using stratified purposeful sampling [39,40], we invited ten first-year and eleven third-year trainees, both male and female. We included first- and third-year trainees from the universities of Nijmegen en Maastricht in order to make the findings applicable to other similar training situations. Our research team included one educationalist, PhD student (MS) and three experienced researchers and educators with differing background as general practitioner (AK), methodologist (HM) and psychologist (CvdV).

Between September 2009 and January 2010, one researcher (MS) conducted all the interviews. The interviewer did not know the interviewees before. The interviews lasted about 30 to 45 minutes and were recorded after verbal informed consent was obtained. The order of the questions and depth of the discussion depended on the interviewees’ input. Interviewees were asked to describe a difficult situation in practice and to reflect on why this was difficult. When it turned out that it was difficult because of not knowing (enough), interviewees were asked to describe what they did to handle the situation, what they did to keep record of things to learn, how they pursued learning and how they eventually estimated what they had learned. Interviewees were encouraged to reflect on their experience in various situations. Interviewees were asked to describe and reflect on the role of their supervisor, mentors and peers in this respect. Furthermore interviewees were asked to describe experienced factors promoting or inhibiting their regulation and learning. The interview topics were created by one researcher (MS) based on theoretical steps of self-regulation applied to GP-training and discussed and refined in the research team. Two pilot-interviews were conducted. The interview topics are given in Additional file 1: Appendix. The participating trainees were informed that the data would be used for educational research purposes only and received a gift coupon for their participation.

## Ethical approval

The study was approved by the Ethical Review Board of the Dutch Association for Medical Education. Anonymity was guaranteed and participation was voluntary.

## Data analysis

The interviews were audio-recorded and transcribed. Data analysis in accordance with a phenomenologic approach contains the steps 1) reading for a sense of the whole, 2) dividing into meaning units, 3) transforming the data and 4) synthesizing the transformed meaning units (describing the structure) [35].

The first two transcripts were read by three of the researchers (MS, HM and AK). Each researcher identified meaning units by codes. Then these two coded transcripts were discussed by the three researchers. The assigned codes seemed to be relevant but too specific to be feasible. This was resolved by regrouping codes in higher-order new codes. Eventually 26 higher-order codes were defined, referring to the process of self-regulation (11 codes), learners expressions on their learning (4 codes), documenting on learning (5 codes), promoting or inhibiting factors (5 codes) and time (1 code). MS and AK then each independently coded five transcripts. Discussion of the coding showed strong agreement. Then the remaining transcripts were coded by one researcher (MS). For each code all relevant text fragments were printed and analyzed independently by MS and AK. During iterative and ongoing discussions relations between codes and recurring themes discerned [39,40]. After analyzing 14 transcripts a pattern of themes and dimensions emerged. The remaining 7 transcripts were used to confirm the themes and dimensions. These 7 transcripts gained no new information so saturation was reached after analyzing 14 transcripts.

During this analytical process MS kept memos and used a logbook to document on coding and analysis. Reflexivity within the research team was adhered by critically questioning and discussing researchers viewpoints on the data and the analysis [41]. Atlas-ti 6.0 software was used for organizing the data.

## Results

**Figure 1:**
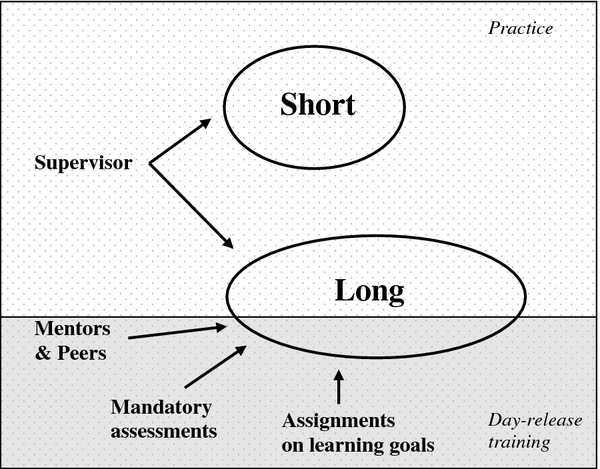
The short and the long loop of self-regulation, and interpersonal and contextual influencing elements.

Two main themes of GP trainees’ self-regulation were identified: 1) self-regulation loops and 2) elements influencing self-regulation. The first theme consisted of two dimensions: a short and a long loop. The second theme included three dimensions: personal, interpersonal and contextual elements. We describe the phenomenon under study by describing the short loop, the long loop and the influencing elements, and illustrate these with quotes [42,43]. Table 1 describes the characteristics of the short and the long self-regulation loop and thereby illustrates the differences between the two loops. Figure 1 illustrates the short and the long loop of self-regulation and the interpersonal and contextual influences hereon. 

**Table 1:** Characteristics of the short and long self-regulation loops derived from 21 interviews with GP-trainees

	**Short loop**	**Long loop**
Context	GP practice	GP practice, occasionally linked to formal training
Duration	Short period of time(during consultation / 1 week at most)	Longer period of time
Monitoring	During consultation signaling lack of knowledge or partial knowledge	During or after consultation by looking back to earlier recurring or complex problems, sometimes based on external assessments or assignments on learning goals
Domains	Minor ailmentsProblems requiring visual observation *(e.g. skin problems*) or immediate action *(e.g. shoulder injection).* Medication/prescriptionMedical Guidelines	Complex problems *(e.g. suspected child abuse, cardiac problems, asthmatic problems,.)* Recurring problems *(e.g. interviewing psychological patients)* Organizational problems *(e.g. time management)* Communication skills
Activities	Singular	Multiple
Assessment	Confidence (based on own feeling, confirmation by patient outcomes or the supervisor)	Confidence (based on own feeling, confirmation by patient outcomes, the supervisor, mentors or peers), sometimes based on results of external assessments
Documenting learning	Memory, post-it notes, patient record or no documentation	Memory, post-it notes, patient record or no documentation, sometimes documentation in learning portfolio

### The short self-regulation loop

The short self-regulation loop was regulated internally and occurred with problems that were relatively easy to solve. It generally lasted one week at most. Self-monitoring occurred when trainees realized they did not know how to solve the problem at hand or only knew a partial solution. This happened during consultations in both the first and third year of training, and was mostly confined to minor ailments and problems that required direct visual observation (e.g. skin problems) or immediate action (e.g. shoulder injections). When trainees realized they knew nothing at all about a problem, they generally asked their supervisor for immediate advice during the consultation.

"“*…for example a skin problem that makes me wonder ‘What is this?’ What I did was ask my supervisor to come and take a look. For well, if I don’t…if I don’t recognize it, I don’t know what to put on it.” (Female, first year, P14)*"

When trainees realized their knowledge was not sufficient but knew where to find the answer, they solved the problem by looking it up during the consultation (e.g. on the Internet, in handbooks) or they prescribed something they thought would help, and made a follow-up appointment. In the meantime they looked for additional information about the case. Usually, this did not require a lot of activities. Most trainees recorded these activities as ‘things to do’ either by making a mental note, a post-it note or a note in the patient record.

"*“….er, this morning, someone with acute stomach pain. What is the best thing I can do right now, for the short term? And then I think ‘I don’t know’. < . > At the time I prescribed something that I knew ‘Well, it will have some effect in any case’ and it is here on a list < . > and I will try and do everything on that list today and that does not always work out but that’s my way of dealing with acute problems and the like.” (Female, third year, P7)*"

The learning activities undertaken by trainees in these cases focused on solving the problem at hand. Generally, the ‘things to do’ were acted upon within a week, but for some trainees they just disappeared from their attention. Most trainees looked up how to solve the problem but did not study underlying factors and mechanisms. Some trainees thought they should study these problems more extensively to gain knowledge for future cases. The trainees assessed improvement of their performance mainly by evaluating their self-confidence during the consultation,and by using guidelines or handbooks.

"*“When I…when I see a patient with these problems and I am sure that I know. When a patient has these problems and I am not afraid to go on, go further and treat or refer, if you’re not afraid, or if you’re sure during the consultation, that means I know.” (Female, first year, P19)*"

"*“…I would say that during a consultation I automatically consider ‘What were the steps again?’ And…then…and…when I have worked that out for myself, I often check whether it was correct what I thought about the steps. And that’s really how I do it, so I use the Guideline to check whether I’m right.” (Female, first year, P12)*"

Clinical outcomes and supervisors opinion during or after consultation contribute to assessment and influence trainees’ self-confidence.

"*“…afterwards he [supervisor] always comes to me and then.well, at first I reviewed almost all patients like ‘I saw this, I saw that, would you also do it that way?’ or ‘is this right?’ And now I only talk about patients when I have a question about them.” (Female, first year, P22)*"

Short-loop learning was not documented in the learning portfolio nor did it involve usage of results of external mandatory assessments as a starting point for learning or to assess competence.

### The long self-regulation loop

The long self-regulation loop was internally regulated but could also be affected by external regulation during the day-release programme. The long loop was generally spread out over a longer period of time, and, unlike the short loop, was used with complex or recurring problems requiring more learning activities. Monitoring occurred during the consultation, when complex problems (e.g. suspected child abuse, cardiac problems) were identified, and also after the consultation when the trainees looked back purposefully over a longer period of time to identify similar recurring patient problems (such as difficulties interviewing patients with psychological problems) or organizational problems (e.g. time management).

"*“…are more structural things, about which I suddenly feel ‘I don’t get enough out of just seeing these patients, I have to do more’. So I have to make a learning goal and I think…well, at least that’s what I understand and that’s also how I see it, how I experience it, that’s the purpose of learning goals, and that’s the way I try to use them to do something more with them.” (Male, third year, P18)*"

Long loop self-regulation of first-year trainees mainly related to communication problems, whereas with third-year trainees it occurred with problems like child abuse, terminal care, cardiac or asthmatic problems or (time) management. Compared to short loop self-regulation, long loop self-regulation was more likely to involve planning of learning activities. Multiple activities were undertaken to solve the problem in question, such as consulting the supervisor, the literature, handbooks or the Internet. The trainees also asked mentors and peers of their day-release group for advice on how to proceed when a problem was difficult to handle or had a strong emotional or personal effect on them.

"*“… a child with an unusual wound and the mother telling a strange story that made me think … it was a burn and I thought of child abuse. I talked about it with my supervisor and he said ‘Well, it’s just once … we won’t do anything about it’, but I kept worrying and I wanted to follow it up. Then I thought ‘well, this….well, I actually don’t like this at all’, so I called the paediatrician who is also treating the child. And because my supervisor did not want to do anything, I thought ‘Well, what should I do?’ So I discussed it in the group and everybody said ‘yes’, including the paediatrician, and all the other trainees in my group said ‘Yes, you really should report this to the Office for the Prevention of Child Abuse. When I reported this to my supervisor like, ‘listen, this is what the paediatrician and my peers advised me, and I am not o.k. with it, so I want to report it’. And he said ‘Yes, yes, all right, you do that, I’ll back you up’. So that's what I did…” (Male, third year, P16)*"

Improvement of performance with regard to these problems was mainly assessed by the trainees in terms of self-confidence based on confirmation by clinical outcomes or by the supervisor, mentors or peers. The long loop was impacted by external regulation when external mandatory assessments revealed shortcomings trainees had not discovered for themselves. In this respect, the trainees especially valued the communication video assessments, as these provided concrete feedback and learning goals, encouraging them to plan learning activities. The trainees also valued the progress meetings, because these enabled them to discuss their progress and learning plans. The trainees made hardly any use of the results of the knowledge tests for their learning, because these tended to vary over time regardless of trainees’ study efforts, and were therefore considered to be less relevant. Several trainees mentioned that the external mandatory assignment to formulate learning goals promoted self-monitoring, encouraging them to consider learning goals they might otherwise not have formulated. Learning as a result of the long self-regulation loop was more likely to be included in the learning portfolio than short-loop learning.

### Facilitating and impeding elements

Elements influencing self-regulation could be divided into three dimensions: personal, interpersonal and contextual elements. The main personal element consisted of trainees’ intrinsic motivation to become a good doctor, which was a strong driver of self-regulated learning.

“And I also think.I feel it's my sense of responsibility that as a GP …I have to keep up, have to know whether…that’s really coming out of myself, and that…I don’t think that that is an idea that is coming from others.” (Male, third year, P3)

Personal elements that were barriers to self-regulation and learning were concentration problems, dealing with too many tasks at the same time or general problems in dealing with negative feedback. Most trainees found themselves active learners, but a few considered themselves passive learners, tending to postpone learning activities or only engaging in them when prompted to do so by others. As for interpersonal elements, trainees reported being stimulated and inspired by their supervisor and the mentors of the day-release programme. Most trainees were very enthusiastic about the way their supervisor encouraged them to find things out and discussed patient problems with them, and by their supervisor’s commitment to their learning.

"*“It helps if…well, I like to be stimulated to learn things, and that may happen during the day-release meeting, because of the planned programme, or because…because someone says ‘Hey, I also wonder how that works, shall we look into it?’ Then it’s a shared question. That stimulates me.” (Female, third year, P5)*"

Unfortunately, some trainees did not experience this type of stimulation and inspiration, which may be due to differences between supervisors. They mentioned a distant or poor personal contact with the supervisor. Two of them experiences even a lack of supervision and felt unable to influence this.

"*“I think I do not get enthusiasm and structure from my supervisor, especially enthusiasm, it’s not motivating. Look, you can also have a supervisor who says ‘Well, go and find out about that’ or ‘maybe we can work on that together’. And, well that just doesn't happen, I have to take the initiative. Well, that doesn’t help.” (Male, first year, P15)*"

Most, but not all, trainees also reported being stimulated by the mentors of the day-release programme. Trainees felt inspired by their peers as a result of sharing problems and similar experiences. The most stimulating contextual element on learning was related to patient encounters, which were not only an incentive for trainees to look things up or plan learning activities, but also had a strong impact on trainees’ retaining knowledge and experiences in memory. Other contextual aspects were characteristics of GP practices, such as the presence of certain types of patients, organizational aspects and a positive working climate.

"*“…it did help, I think, that the practice assistants were willing to support me, it can work against you if the assistant doesn’t want to change the schedule. Luckily it went well this time, so they think along with you. So that helps.” (Female, third year, P1)*"

Factors that were seen as barriers to self-regulation were time pressure, the absence of certain types of patientsdifficulties in planning learning activities,

"*“…and I didn’t see all that many because most of the gynaecological cases are seen by the female GP, and I mostly get patients from my [male] supervisor, so I do not get to place all that many IUDs. Then I did my best to do something < . > It is something we have to be able to do at the end of first year. I want to be able to do it and I did not get to see those patients as a matter of course.” (Male, first year, P9)*"

“Only… it happened quite often that the coaching sessions on Thursdays were cancelled for some reason or other, so that was something, and in the end we rescheduled these sessions, for they were always Thursdays from 5 to 6 pm, and then from 6 to 7 pm, well, then I’m not really motivated, she also wanted to go home, so now we meet in the morning, at the end of the morning, and yes, that’s much better, and there’s time reserved for it, that's quite nice.” (Male, third year, P15)

and trainees' inability to change practice routines that hindered their learning.

A contextual aspect relating to the day-release programme was the positive atmosphere, although some trainees felt this could be improved. Some trainees mentioned difficulties combining tasks related to work and private life.

## Discussion

With this study we wanted to gain more insight in how trainees regulate their learning in practice. The analysis of the interviews revealed that self-regulation of learning occurred in a short and a long loop, of which only the latter was influenced by external regulation like mandatory assessments or assignments to formulate learning goals. Self-regulation and learning were driven by trainees’ strong intrinsic motivation to become good doctors, prompted by others and influenced by organizational and educational elements in the training context. To elucidate our findings we will discuss the two self-regulation loops from the perspective of learning theory and the three elements influencing self-regulation from the perspective of Self-Determination Theory (SDT) [44-46].

### Learning theory: monitoring and assessment

The learning we found in the short and the long loop can be seen as informal learning. Since learning in both self-regulation loops occured in reaction to an incidental learning need, with the long loop also involving planned learning, the short and long self-regulation loops appear to fit the types of reactive and deliberative learning as described by Eraut [47-49]. Reactive learning, according to Eraut, happens spontaneously when an unexpected situation makes the learner aware that something must be learned. Deliberative learning is similar to reactive learning but differs in that it includes planning of learning activities.

Both loops involved self-monitoring. Although our study did not focus on reflection, the analysis revealed that trainees reflected on their actions both during and after consultations, suggesting that trainees' self-monitoring may refer to Schön's ‘reflection-in-action’ (short loop) and 'reflection-on-action’ (long loop) [5].

Learning orientations may influence the way trainees plan and organize their studying [7]. Learning in both loops originating from practice situations is associated with situational orientation. Learning in the long loop initiated form external regulation, like mandatory assignments, may be associated with course specific orientation.

In both loops trainees based their self-assessments on their confidence in their ability to perform competently. The variation among trainees in the extent to which they asked their supervisor to confirm their self-assessed competence suggests that trainees should be encouraged to seek more external information to confirm their self-confidence by consulting external sources, such as supervisors and mentors [12,22,50]. This is important because self-assessment undertaken as an individually conducted internal activity has little accuracy, especially for those with the least proficiency [51]. This implies an active and critical role from supervisors, as in a work-based learning environment it is important for supervisors to encourage trainees to engage in critical reflection and be a role model in this respect [52,53]. Trainees in our study variably used external assessments for learning. This may be explained by the level of the mandatory assessments. In Miller's assessment pyramid, assessments range from the lowest 'knows' level, via the ‘knows how’ and ‘shows how’ levels to the highest ‘does’ level [27,54]. The communication video assessments are aimed at the ‘does’ level and provide specific and easily applicable feedback. The knowledge progress tests, however, are aimed at the ‘knows’ and ‘knows how’ levels. Trainees see feedback from these tests as having limited relevance to their performance. Further research should investigate ways of promoting the effectiveness of external assessments as well as ways of assessing practice performance in both loops that provide external information to confirm trainees’ self-confidence or in any other way scaffold their competence.

In conclusion, it is important to know how learning in the short loop takes place. This learning may not be visible to others. Therefore supervisors can play an important role in guiding learning and assessment in the short loop as they are the ones close to trainees. Formal learning activities, like learning portfolios or assessments, give opportunities to encourage trainees in monitoring, employing learning activities and assessment [23]. As already known medical practice offers a powerful setting for informal learning [23,47,48]. However, it should be noted that relying on informal learning may involve the risk of learning and maintaining inadequate competencies, habits or behaviours [52]. This stresses the importance of critical reflection, aimed at one’s attitudes and frames of references on (implicit) habits, behaviours, professional acting and professional learning [52].

### Influencing elements and the Self-Determination Theory

We found influencing elements on personal, interpersonal and contextual level. The Self-determination theory (SDT) helps us to explain them and identify potential relationships between them [44-46]. According to SDT, human behaviour is determined by motivation, varying along a continuum from lack of motivation via extrinsic motivation to intrinsic motivation [44,45,55]. People driven by high internal motivation are more likely to achieve their goals than people driven by high external motivation [44,46,55]. Furthermore, SDT describes that goal pursuit and attainment are strongly related to the extent to which people are able to satisfy three basic psychological needs: (a) the need for autonomy, (b) the need for competence and (c) the need for relatedness to others and to the social environment [44,45,55]. People are more likely to adopt activities that are valued by relevant social groups when they feel efficacious with respect to those activities (*need for competence).* Internalization is also facilitated when the context supports autonomy, allowing the learner to feel competent, related and autonomous *(need for autonomy).*Behaviours prompted, modelled or valued by significant others to whom someone feels (or wants to feel) attached or related (*need for relatedness*) are more likely to be internalized.The influencing elements we found (on personal, interpersonal and contextual level) are related to intrinsic motivation and to the need for competence, autonomy and relatedness. Trainees reported a strong internal motivation to become good doctors. This motivation was a strong driver of self-regulated learning when they identified shortcomings in their performance, thereby fulfilling their need for competence and autonomy. To gauge their competence trainees appeared to rely mostly on self-confidence. Self-confidence may be based on self-efficacy beliefs resulting from judgements of one’s ability to deal with different situations [56-58]. There is evidence that self-efficacy beliefs contribute significantly to motivation and performance [56]. Trainees also sought to varying extent confirmation from supervisors, mentors and peers. However, external mandatory assessment, the knowledge tests in particular, played a less prominent role in trainees’ self-assessment of their competence. Apparently, external assessments fail to meet the psychological conditions that are conducive to enhancement of motivation. This suggests that supervisors and mentors should promote the use of assessments by actively alerting trainees to the relevance of this feedback for their performance in practice.

Trainees also reported the influence of others on their self-regulation and learning. Following SDT this may be explained in terms of fulfilment of the need for relatedness. Supervisors were found to be especially important as role models and as someone trainees could feel related to. These findings correspond with the literature on supervisor’s role in medical education [59,60].

Finally, trainees reported the influence from the workplace. The contextual elements like patient encounters, working climate, organizational and educational features and time pressure, are also described in the literature about workplace based learning, where the quality of the workplace and its educational aspects influence opportunities for and the quality of learning [23,52,61-64]. According to SDT context is important in supporting feelings of relatedness, competence and autonomy.

In summary, SDT offers explanations of the way trainees’ self-regulation is driven by intrinsic motivation, depending on the extent to which trainees’ needs for competence, autonomy and relatedness are met. The context of training and the actors in it are important in supporting these needs.

### Differences between first and third years trainees

We only found differences in the kind of problems trainees mentioned in the long loop. In the long loop first year trainees reported mainly problems with communication skills. An explanation may be that an adequate mastery of these skills is prerequisite for working in general practice. Third year trainees mentioned more complex problems in the long loop. May be they are more likely to have such problems assigned to them than first year trainees.

The absence of differences in the short loop may be explained by the wide variety of patient problems in general practice making it impossible for trainees, even at advanced stages of training, to know everything about all types of frequently presented patient problems.

First and third year trainees did not differ in the influencing elements.

### Strengths and weaknesses of this study

The results of this study of trainees’ self-regulation showed that medical practice is indeed a powerful learning environment, but also highlighted the complexity of the components and relationships within this environment [65]. A weakness of the study may be that the exclusive focus on trainees' self-regulation precluded in-depth examination of potentially important influencing elements like the relationship between trainee and supervisor and the role of peers. Another weakness may be the fact that we exclusively used interviews to gather data. Observation of actual practice and analyzing portfolios (regarding the long-loop) may refine viewpoints on self-regulation. Also, since the study was limited to trainees' perceptions, no conclusions can be drawn regarding the supervisors’ views on trainees’ self-regulation. A strength of this study is the phenomenological qualitative approach, which enabled in-depth exploration of trainees’ individual views and experiences and resulted in the discovery of patterns.

Further research should focus on learning in the short and long loop and on how formal learning (e.g. external assessments, learning portfolio’s), people (e.g. supervisor, mentors) and context (e.g. educational quality, working climate) can contribute to self-regulation and thereby to learning. Further research on behavioural measures relating to learning and supervision of trainees and supervisors may strengthen the results found in this study.

## Conclusion

GP trainees appeared to use a short and a long self-regulation loop, with external regulation playing a role in the long loop only. Self-regulation enables trainees to fulfil their needs for autonomy and competence. To compensate for weaknesses of self-assessment, educational programmes should promote self-directed assessment seeking and critical reflection. External assessment remains also necessary, however, to ensure that trainees learn appropriate competencies in both loops, and special attention should be paid to encouraging trainees to make use of the results of external assessments for their learning in both loops. Efforts should therefore be made to support trainees in using external feedback. The quality of supervisors and the workplace can contribute to trainees’ self-regulation by fulfilling their need for relatedness, competence and autonomy.

## Competing interests

The authors declare that they have no competing interests.

## Authors’ contribution

All authors contributed to the conception and design of this study and the analysis and interpretation of data. MS conducted the acquisition of data and drafted the manuscript. All authors participated in critical revision of the manuscript and read and approved the final manuscript.

## Acknowledgements

This study was supported by funds from the SBOH, a Dutch foundation and formal employer of GP trainees.

The authors gratefully acknowledge the GP-trainees who participated in this study, Henk Mokkink (HM) for his contribution during design and data-analysis, Bas Maiburg and Els Pelgrim for their advice during this study, Mereke Gorsira for assistance in manuscript editing and Elke Butterbrod for assistance in transcribing.

## Pre-publication history

The pre-publication history for this paper can be accessed here:

http://www.biomedcentral.com/1472-6920/12/67/prepub

## Supplementary Material

Additional file 1 **Appendix.** Interview topics used in 21 semi-structured interviews with first- and third-year GP-trainees. Click here for file
