# Supplementary material for: How do postgraduate GP trainees regulate their learning and what helps and hinders them? A qualitative study
Source: BMC Med Educ. 2012 Aug 6;12:67. doi: 10.1186/1472-6920-12-67 (PMC3479408; doi:10.1186/1472-6920-12-67)
Supplement: Additional file 1 — Appendix. Interview topics used in 21 semi-structured interviews with first- and third-year GP-trainees. [file 1472-6920-12-67-S1.doc]

***Appendix 1: Interview topics used in 21 semi-structured interviews with first- and third-year GP-trainees***

| Can you describe a difficult situation in practice? What did you do to handle the situation? How did you keep track of the things you had to learn from this situation? What kind of activities did you undertake to learn these things? How did you know you had learned what you wanted to learn? Is the learning process you just described illustrative of the way you usually learn or do you also learn in other ways? Did you consult others when you were learning?  Did you use your portfolio when you were learning in the way you just described? For what learning goals do you consult your supervisor or your mentors at the institute?  To what extent do external mandatory assessments inform you about potential learning goals? How does the day-release programme contribute to your learning? How would you describe your own role in your learning?  What helps you in your learning? What hinders you in your learning? |
| --- |
